# Being Hungry Affects Oral Size Perception

**DOI:** 10.1177/2041669518777513

**Published:** 2018-05-22

**Authors:** Parker Crutchfield, Vanessa Pazdernik, Gina Hansen, Jacob Malone, Molly Wagenknecht

**Affiliations:** Western Michigan University Homer Stryker M.D., School of Medicine, Kalamazoo, MI, USA; Missouri School of Dentistry and Oral Health, A.T. Still University, Kirksville, MO, USA; Department of Research Support, A.T. Still University, Kirksville, MO, USA; Missouri School of Dentistry and Oral Health, A.T. Still University, Kirksville, MO, USA

**Keywords:** oral perception, hunger, predictive coding

## Abstract

Oral size perception is not veridical, and there is disagreement on whether this nonveridicality tends to underestimate or overestimate size. Further, being hungry has been shown to affect oral size perception. In this study, we investigated the effect of hunger on oral size perception. Overall, being hungry had a small but significant effect on oral size perception and seemed to support that oral size perception tends to underestimate the size of objects. Both hungry and sated participants tended to underestimate the size of intraoral objects, but hungry participants underestimated to a significantly lesser degree. Unlike previous research, this tendency was independent of the order and number of assessments of size. We, therefore, offer a novel explanation for these findings: Oral size perception is modulated by a hierarchy of Bayesian predictions, and being hungry changes the priors in these predictions.

## Introduction

Of the various perceptual capacities humans are endowed with, oral perception is among the least frequently researched. With the exception of research on taste and texture of foods, we know comparatively little about perception by way of the oral cavity. However, properly functioning perceptual abilities of the oral cavity are critical, especially since they mediate chewing and swallowing, the only nonmedical means of ingesting life-sustaining nutrition.

Much of the research conducted on the perceptual capacities has focused on oral size perception and the variables that affect it. There is univocal evidence that oral size perception is commonly nonveridical. Some studies (Anstis, 1964; Anstis & Loizos, 1967; La Pointe, Williams, & Hepler, 1973) found that when perceiving the size of holes with the tongue, the holes are consistently overestimated. In some cases, this overestimation was greater for the smaller holes in the target object, while in others the overestimation was greater for the larger holes. However, others (La Pointe et al., 1973) found a slight underestimation for smaller holes but overestimation for larger holes.

In another study (Dellow, Lund, Babcock, & Rosendaal, 1970), instead of exploring holes with the tongue, participants assessed the size of intraoral cylinders. Most of the participants’ erroneous assessments were overestimations. More recently, participants assessed the size of both holes and pegs with their tongues and fingers (Bittern & Orchardson, 2000; Melvin & Orchardson, 2001). For most of the holes and pegs, participants overestimated the size. For a small number of hole and peg sizes, however, participants underestimated their size.

Some evidence suggests that oral size perception is commonly nonveridical and that the nonveridicality is mediated by other variables. For example, in one study (Engelen, Prinz, & Bosman, 2002), participants assessed the size of intraoral spheres by visually matching them to those on a reference set. Some of the participants wore a palate covering to test the influence of the hard palate on oral size perception. In both groups, participants underestimated the smaller spheres and overestimated the larger spheres, though for some wearing the palate covering the nonveridicality was diminished. Some researchers (Crutchfield, Mahoney, Pazdernik, & Rivera, 2016) found underestimation of intraoral spheres using visual matching, while others (Topolinski & Turk Pereira, 2012) found size underestimation using digital matching, where participants matched the size of an intraoral cylinder with a reference set using their fingers.

The severity of the nonveridicality of oral size perception is also subject to other psychological variables. Crutchfield et al. (2016) found that the nonveridicality of oral size perception could be manipulated by priming participants with differently sized spheres assessed with a different sense modality, which showed a priming effect as well as a cross-modal effect on oral size perception. They also found both overestimation and underestimation. Preliminary evidence also suggests that the time spent orally manipulating an object influences whether the object is overestimated or underestimated (Tomita, Murakami, Takahashi, Ooka, & Hironaka, 2017). Topolinski and Turk Pereira (2012) found that satiety may influence oral size perception. In their study, compared with participants who were not hungry, hungry participants’ oral size estimates were larger, though both groups underestimated oral size. Their explanation for the observed effect was that hunger increased oral sensitivity; the effect of being hungry diminished as participants repeated matching tasks, which desensitized the oral cavity as they did so.

Other studies have produced evidence disconfirming candidate explanations of oral size perception and its nonveridicality (Bittern & Orchardson, 2000; Engelen et al., 2002; Melvin & Orchardson, 2001). The evidence that hunger can influence oral size perception is of particular interest, since it suggests an explanation for the apparent disposition to nonveridical oral size perception. To test for an effect of hunger on oral size perception, we sought to examine the effect satiety has on oral size perception and whether this effect was more prevalent for visual or digital matching tasks.

## Methods

This study was reviewed by the institutional review board of A.T. Still University, Kirksville, and exempted from subsequent review, and all participants provided written consent to their participation after receiving information regarding the risks, benefits, and commitments of such participation. Participants were recruited by e-mail. The sampling frame consisted of all enrolled medical and dental students at A.T. Still University in Kirksville, Missouri.

Once enrolled, all participants were randomized into a control or experimental group, then put into blocks of four (two in each group) according to the day they were available to participate. Each block arrived at the study site at noon for their session. Data were collected during 16 different sessions. Participants in the control group were given no instructions regarding their intake of food and drink. Participants in the experimental group were instructed to have a normal dinner and breakfast, but to not eat anything after 8:00 a.m. and to drink only water between 8:00 a.m. and noon.

Once participants arrived at the study site, those in the control group were given a large lunch consisting of a large sandwich, a small bag of chips, and a large cookie. While the control group ate, those in the experimental group entered separate observation rooms to perform the tasks. Prior to performing the tasks, all participants rated their level of hunger (preassessment hunger survey) on a scale from 1 to 5, with 1 corresponding to *being full* and 5 to *being very hungry*.

Participants were tasked with matching intraoral stainless steel spheres with a visual and digital reference set. Four sizes of spheres were assessed, but eight sizes of spheres were included in the reference set (Table 1), so that it was possible to overestimate or underestimate on any assessment. The reference set was the set of spheres affixed to a clear acrylic plate. Participants were blindfolded and then offered a cup containing a sphere. Without touching or looking at the sphere, they placed the sphere in their mouth. They then matched the intraoral sphere to the reference set. If the task was to digitally match it, they kept the blindfold on and used their fingers to explore and match the spheres. If the task was to visually match it, they removed the blindfold and visually matched the intraoral sphere to the reference set. Once a participant matched the intraoral sphere to a sphere on the reference set, the size of the selected sphere was recorded. All four sized spheres were assessed digitally and visually, resulting in eight matching tasks for each participant. The order of the eight matching tasks was completely randomized to eliminate any ordering effect.

**Table 1.** Size of Spheres Used in Inches and Millimeters.

	Sphere size	
Assessment used	1/16 in.	mm
No	1	1.6
No	2	3.2
Yes	3	4.8
Yes	4	6.4
Yes	5	7.9
Yes	6	9.5
No	7	11.1
No	8	12.7

Once the participants in the hungry group completed the tasks, they were offered a lunch, while the sated control group completed the tasks.

A χ^2^ test and *t* test were used to compare gender and ages between groups, respectively. Frequencies and percentages were calculated for the preassessment hunger survey and veridicality in the matching tasks. A repeated measures analysis of variance (ANOVA) was used with the outcome variable as the difference between the estimate (participant’s selected reference sphere) and the actual (intraoral sphere) size in units of 1/16 in. diameter or spheres (i.e., sphere 3, 4, 5, or 6; Table 1). Negative values indicated underestimation and positive values indicated overestimation. The between-groups factor was the group (control or experimental) and the repeated factors were the assessment modality (digital or visual) and the intraoral sphere size (3/16, 4/16, 5/16, or 6/16 in. diameter). A second ANOVA model including the three previously stated set of factors plus an additional repeated factor, the order of the assessment, was used to test for an interaction between groups and the first and last assessment while controlling for the assessment modality and the intraoral sphere size. Following ANOVA, pairwise comparisons were made with Bonferroni adjustment to control the type 1 error rate. Estimates and means are provided with associated confidence intervals (CIs). Statistical significance was defined as *p* < .05. Analyses were conducted using SAS version 9.4 (SAS Institute Inc., Cary, NC).

## Results

Sixty-four people participated in the study: median age, 26 years; range, 22 to 38 years; 42 (66%) females. Thirty-two participants were randomized to each group. Age and gender were balanced between the control and experimental groups: median age, 25 years and 26 years, respectively; 20 (62%) females and 22 (69%) females, respectively (both *p* > .59).

For the preassessment hunger survey, two participants did not provide a rating—one from each group. All remaining participants in the control group rated themselves as either *somewhat* (*n* = 6, 19%) or *very full* (*n* = 25, 81%). All remaining participants in the experimental group rated themselves as either *somewhat* (*n* = 14, 45%) or *very hungry* (*n* = 17, 55%).

Overall, almost half (*n* = 237, 46%) of assessments were veridical (Table 2). Of the 54% of assessments that were nonveridical, 69% (*n* = 190) were underestimation (37% of all assessments). Overestimation accounted for 17% (*n* = 85) of all assessments.

**Table 2.** Veridicality Distribution and Differences Between the Estimated Sphere Size and Actual Sphere Size of All Assessments for Both Groups and Assessment Modalities.

Size (1/16 in.)	Assessment modality	Group	Underestimate	Veridical	Overestimate	Difference (estimate − actual) [95% CI]	*p*
3	Digital	Control	7 (22%)	18 (56%)	7 (22%)	0.00 [−0.25, 0.25]	>.99
		Experimental	5 (16%)	16 (50%)	11 (34%)	0.25 [0.00, 0.50]	.048
	Visual	Control	7 (22%)	24 (75%)	1 (3%)	−0.19 [−0.44, 0.06]	.14
		Experimental	4 (13%)	22 (69%)	6 (19%)	0.13 [−0.12, 0.37]	.32
4	Digital	Control	12 (38%)	19 (59%)	1 (3%)	−0.41 [−0.65, −0.16]	.001
		Experimental	13 (41%)	12 (38%)	7 (22%)	−0.16 [−0.40, 0.09]	.22
	Visual	Control	25 (78%)	7 (22%)	0 (0%)	−0.97 [−1.22, −0.72]	<.001
		Experimental	23 (72%)	8 (25%)	1 (3%)	−0.78 [−1.03, −0.53]	<.001
5	Digital	Control	9 (28%)	17 (53%)	6 (19%)	−0.09 [−0.34, 0.15]	.46
		Experimental	9 (28%)	17 (53%)	6 (19%)	−0.09 [−0.34, 0.15]	.46
	Visual	Control	26 (81%)	5 (16%)	1 (3%)	−0.88 [−1.12, −0.63]	<.001
		Experimental	14 (44%)	13 (41%)	5 (16%)	−0.31 [−0.56, −0.06]	.01
6	Digital	Control	4 (13%)	15 (47%)	13 (41%)	0.28 [0.03, 0.53]	.03
		Experimental	8 (25%)	10 (31%)	14 (44%)	0.16 [−0.09, 0.40]	.22
	Visual	Control	13 (41%)	18 (56%)	1 (3%)	−0.47 [−0.72, −0.22]	<.001
		Experimental	11 (34%)	16 (50%)	5 (16%)	−0.22 [−0.47, 0.03]	.08
Total			190 (37%)	237 (46%)	85 (17%)		

*Note.* For the difference between the estimate and actual sphere size, a value of .5 indicates overestimation of one half of a sphere. A value of −.5 indicates underestimation of one half sphere. Underestimate, veridical, and overestimate are reported as *n* and percentage. CI = confidence interval.

**Figure 1.**
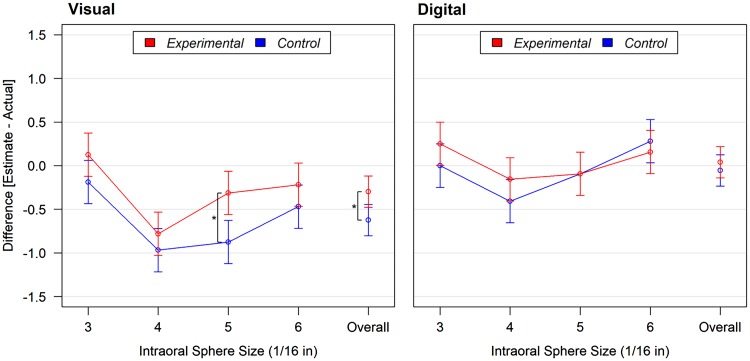
Comparisons between the control and experimental groups on differences between the visual (left panel) or digital assessment (right panel) estimate and intraoral sphere size by intraoral size and overall. Overall category is the mean difference between the estimate and actual size across all four different intraoral sphere sizes. *p ≤ .01.

A significant interaction between groups and assessment modality was found on the outcome variable, the difference between the estimate and actual size (*p* = .02; Figure 1). Although assessments overall tended toward underestimation, participants in the experimental group underestimated the size of the spheres to a lesser degree when visually matching the intraoral sphere. For all visual assessments and compared with the control group, participants in the experimental group perceived the size of the spheres to be 0.33, 95% CI [0.07, 0.58] spheres bigger (*p* = .01), which amounted to 1/2 mm difference. The largest difference between groups was observed for the intraoral size of 5/16 in.; participants in the experimental group perceived the size to be 0.56, adjusted 95% CI [0.12, 1.01] spheres bigger (adjusted *p* = .007). Between groups, there was no significant difference in digital assessment of oral size (*p* = .47).

Table 2 presents the differences between the estimate and actual size for each assessment. For the experimental group, three assessment types were statistically significant, and two of these had a perceived size that was underestimated. For the control group, most assessments were statistically significant, and all but one size were underestimated.

When testing for a difference between perceived size of the first sphere and last sphere assessed, we found that the effect of hunger on oral size perception was greater (*p* = .10) for the last sphere assessed (control, − 0.44 vs. experimental, −0.17; difference, −0.26) than it was for the first sphere assessed (control, −0.24 vs. experimental, −0.31; difference, 0.07).

## Discussion

The results of this study indicated that oral size perception is frequently nonveridical. This finding builds on previous studies that support the same inference. Further, when oral size perception is nonveridical, this study suggested that nonveridicality is usually an underestimation. Sixty-nine percent of all nonveridical estimates were underestimations. This bias was stronger for those participants who were sated when estimating the size of the spheres. This finding also coheres with previous studies that found a bias toward underestimation (Bittern & Orchardson, 2000; Crutchfield et al., 2016; Dellow et al., 1970; Engelen et al., 2002; Melvin & Orchardson, 2001; Tomita et al., 2017; Topolinski & Turk Pereira, 2012). However, it conflicts with other research that found a bias toward overestimation (Anstis, 1964; Anstis & Loizos, 1967; Dellow et al., 1970; La Pointe et al., 1973).

One explanation that reconciles this apparent conflict is that the studies which show a bias toward underestimation have similar study designs and those that show a bias toward overestimation also have similar designs. Thus, the indicated biases may be dependent on the study design. For instance, those studies that showed underestimation used intraoral objects and those that showed overestimation did not. The design of this study required that participants place small round objects in their mouths to assess size. These spheres approximated the size of pieces of food. Presumably, human oral perceptual capacities may be generalizable from observations about how the size of small intraoral objects are perceived. But there is some tenuous evidence that obvious nonfood items are perceived differently than similarly sized food items (Brogden, Sinclair, & Almiron-Roig, 2009).

Previous research suggests that being hungry can influence oral size perception (Topolinski & Turk Pereira, 2012). This study also suggested that hunger can influence a person’s perception of the size of intraoral objects. The participants who were in the hungry experimental group underestimated the size of the spheres to a significantly lesser degree than those in the sated control group. However, the effect of hunger on oral size perception was only observed for visual matching tasks; no effect was observed for the digital matching tasks. When compared with being sated, being hungry makes things in the mouth seem orally bigger. The magnitude of the difference was small, but the perceptual difference may be large. For instance, even a tiny raspberry seed stuck in the gums or in the cusps of a tooth can feel quite large. Small measurable differences do not translate to small perceptual differences.

Topolinski and Turk Pereira (2012) attribute their finding of hunger influencing oral size perception to the desensitization of the oral cavity, partly because they found that the difference in size estimation between those who were hungry and those who were not decreased with repeated assessments. Their explanation is that food deprivation also deprives the oral mucosa, leading to greater oral sensitivity (Zuckerman & Cohen, 1964). As participants in their study continued assessing the size of intraoral objects, the oral cavity desensitized, leading to less of a difference between those who were hungry and those who were not. In this study, we tested for a similar effect and found none. If anything, the effect of hunger was greater for the last sphere assessed, given that the differences between the sated and hungry groups were greater for the last sphere assessed than they were for the first sphere assessed. If differences in size perception were attributable to initial sensitization of those who were hungry, we would expect the differences in the final sphere assessed to be smaller than those in the first sphere assessed, no matter the size of the sphere. Therefore, the effect hunger has on oral size perception was not evidently mediated by sensitivity of the oral cavity.

A potential explanation for the observed overall tendency toward underestimation and the observation that hungry people orally perceive things as bigger than nonhungry people may be that the mind operates according to hierarchy of Bayesian inferences and that one’s hunger modulates the probabilities that interact with oral size perception. This model of perception and cognition conceives of the mind as a prediction machine, where the predictions are governed by Bayes’ Theorem. According to this predictive model, higher levels in the hierarchy pass predictions (Bayesian priors) down through the lower levels. These expectations exert top-down influence on what one perceives. At the same time, lower levels in the hierarchy code sensory information and pass it up. As they do so, the incoming information (Bayesian posteriors) updates the priors if the priors do not match the posteriors. The result is a continuous series of inferences, the premises of which are the top-down predictions and the bottom-up sensory input.

Among the virtues of this predictive coding model of the mind is that it is economical. Only errors—mismatches between what’s predicted and what’s detected—are passed up the hierarchy. Other alleged virtues are that it is powerful and able to explain a wide range of phenomena, that it is empirically well supported, and that it presents a simple, unified view of the mind (Clark, 2013; Hohwy, 2013). This predictive coding model of the mind may explain the observations that oral size perception tends toward underestimation and that hungry people perceive things as bigger.

When sated, there is little value in ingesting intraoral objects. In particular, given that one is sated, small intraoral objects may be more likely to be hazardous than nutritious, and so to minimize hazard and discourage further ingestion, the priors may be set such that higher probability is given to smaller-sized intraoral objects. The risk of ingesting relative to the benefit changes the priors. Thus, when one is sated, the size of intraoral objects is often underestimated unless compelling bottom-up sensory inputs update prior predictions. But being hungry may change the priors involved in making predictions about the nature of the intraoral object. Given that one is hungry, intraoral objects are more likely to be nutritious. Thus, no strong prediction of size is made. When one intraorally encounters a small object, the prior underlying the disposition for underestimation is attenuated, making it more likely that one will ingest the object and satisfy the need to eat. When hungry, the risk of ingesting relative to its benefit attenuates the predictions responsible for underestimation.

This explanation implies that the detected size of the intraoral object interacts with the brain’s underlying assumptions about what is likely to be encountered. Small objects are underestimated to discourage ingestion. This underestimation is attenuated in hungry people to encourage ingestion. By the same token, for sated people, larger objects would be overestimated since they could be potentially hazardous (e.g., may lead to choking), but this overestimation would be attenuated in hungry people. In this study, all intraoral objects were small, so we did not test this prediction. But previous research has indicated a size-dependent oral size illusion where the sizes of larger intraoral objects were overestimated and small objects underestimated (Crutchfield et al., 2016). In that study, the level of hunger was uncontrolled, but the results provide indirect support for the observed attenuated underestimation of size of hungry participants in this study.

Previous research (Crutchfield et al., 2016) also shows that oral size perception can be influenced by differently sized spheres perceived immediately prior to perception of an intraoral sphere. The predictive coding model offers an explanation for this observation: The perceived size of a priming sphere changes the expectations—the priors—that exert the top-down influence on the perception of the intraoral sphere.

This explanation, however, does not yet account for the observation that the effect only occurred when the task was to match the intraoral sphere visually to the reference set. Being hungry had no effect on oral size perception when the task was to match the intraoral sphere to the digital reference set. The only way the predictive coding model could account for these different observations is if the priors for the visual matching were set differently from those of the digital matching. Further, the priors influencing perception by way of the fingers must be similar to those influencing perception by way of the oral cavity.

That the priors influencing perception by way of the fingers were similar to those influencing perception by way of the oral cavity is supported by the notion that oral perception and digital perception are both instances of haptic perception. That oral perception is a token of haptic perception is commonly accepted. If it is true that oral perception and digital perception are both tokens of haptic perception, then it is plausible that they are influenced by the same prior (which may occur at the level of the type).

But for a difference in priors between modalities to account for the observed difference in oral size perception, it must also be true that hunger affects those priors influencing vision differently. Kahrimanovic, Bergmann Tiest, and Kappers (2010) showed that the strategies used in haptic perception of objects are different from those used in visual perception. Specifically, haptic perception may rely more heavily on surface area, whereas vision relies primarily on shape properties. Furthermore, which shape properties are used varies among the participants. They also showed that for haptic-visual bimodal volume perception, haptic input (surface area) has a greater influence on perceived size. Thus, the priors influencing visual perception of size (volume) may be more susceptible to influence from other modalities or other variables. This greater susceptibility to influence from other variables, when combined with the idea that digital and oral perception are both haptic perception, may explain why hunger had an effect on visual matching but not on digital matching

The above explanation is speculative, given the relatively thin evidence from this study, especially since it is easy to attribute priors post hoc. But the explanation suggests avenues of future research, such as research on how hunger affects haptic perception and research on the comparable susceptibility of visual size perception to other types of influence, cross-modal and otherwise.

One limitation of this study has already been noted: We only tested the size of four spheres, all of which were small. Additional research should test the prediction that hungry people will overestimate the size of larger objects to a lesser degree than sated people. A second limitation is that the temperature of the spheres was uncontrolled. As such, the spheres used in the second assessment of a particular size may have been warmer than in the first assessment of that size, which may have influenced the results. However, Tomita et al. (2017) tested for such an effect and found none. Nevertheless, if the attenuated underestimation were due to temperature, one would expect that effect to be less in the final assessment, which is contrary to what was observed in this study. Another limitation is related to the ingestion of food. Specifically, if oral size perception is inferentially linked to hunger and the ingestion of food, then oral size perception may be affected differently when an intraoral object is actually food. We only used stainless steel spheres for the sake of variable control. Finally, the duration of hunger for the experimental group was only 4 hours. Longer durations of hunger may have a greater influence on oral size perception.

## Conclusion

Abundant research indicates that oral size perception is frequently nonveridical. More recently, research suggests that oral size perception, when nonveridical, is frequently underestimated. However, this underestimation can be influenced by other factors, including the hunger level of the perceiver. In this study, we tested the effect of hunger on the size perception of intraoral objects and found that being hungry had a small but significant effect on oral size estimation in visual matching, but not in digital matching, when compared with being sated.
